# Sensitization to storage proteins in peanut and hazelnut is associated with higher levels of inflammatory markers in asthma

**DOI:** 10.1186/s12948-020-00126-5

**Published:** 2020-06-23

**Authors:** Jennifer Johnson, Andrei Malinovschi, Jonas Lidholm, Carl Johan Petersson, Lennart Nordvall, Christer Janson, Kjell Alving, Magnus P. Borres

**Affiliations:** 1 Department of Women’s and Children’s Health, Uppsala University, Uppsala, Sweden; 2 Department of Medical Sciences, Clinical Physiology, Uppsala University, Uppsala, Sweden; 3 Thermo Fisher Scientific, Uppsala, Sweden; 4 Department of Medical Sciences, Respiratory, Allergy and Sleep Research, Uppsala University, Uppsala, Sweden

**Keywords:** Asthma, Allergy, Specific IgE, Molecular allergy diagnostics, Food allergen components, Hazel nut, Peanut, Sensitization

## Abstract

**Background:**

Sensitization to peanuts and hazelnuts is common among young asthmatics and can be primary or a result of cross-reactivity. Sensitization as a result of cross-reactivity to birch pollen is typically associated to tolerance or mild and local symptoms upon intake of peanut or hazelnut.

**Aim:**

The aim of this study was to investigate relationships between IgE antibody responses against peanut and hazelnut components, airway and systemic inflammation markers, lung function parameters and reported food hypersensitivity in a cohort of asthmatic children and young adults.

**Methods:**

A population of 408 asthmatic individuals aged 10–35 years were investigated. Information on hypersensitivity symptoms upon intake of peanut or hazelnut were recorded in a standardized questionnaire. Fraction of exhaled nitric oxide (FeNO), blood eosinophil count (B-Eos), spirometry, methacholine challenge outcome and IgE antibodies to peanut and hazelnut allergens were measured by standard clinical and laboratory methods.

**Results:**

Subjects sensitized to any of the peanut (Ara h 1, 2 or 3) or hazelnut (Cor a 9 or 14) storage proteins were significantly younger (17.6 vs 21.2 years), had higher levels of FeNO (23.2 vs 16.7 ppb) and B-Eos (340 vs 170 cells/mcl) than those displaying only pollen-related cross-reactive sensitization. Levels of FeNO correlated with levels of IgE to storage proteins in children, but not in adults. Levels of B-Eos correlated with levels of IgE to all allergen components investigated in children, but only to levels of IgE to storage proteins in adults. Anaphylaxis and skin reactions upon intake of peanuts or hazelnuts were more often reported among subjects sensitized to the respective storage proteins than among those with only pollen-related cross-reactive sensitization. As compared to peanut, hazelnut was more often reported to cause gastrointestinal symptoms and less often oral cavity symptoms.

**Conclusions:**

Sensitization to peanut and hazelnut storage proteins was associated with higher levels of inflammation markers and food hypersensitivity symptoms in this population of subjects with asthma.

## Background

Sensitization to a particular allergen source can be either primary or secondary, the latter occurring as a result of cross-reactivity to proteins from a different allergen source but with similar molecular structures. The major peanut allergens are the storage proteins Ara h 1, Ara h 2 and Ara h 3 [1–4]. IgE against Ara h 2 is the most important predictor of clinical peanut allergy [2–9] and may be used to reduce the need for food challenges. IgE recognition of Ara h 8 occurs as a result of cross reactivity with the birch pollen allergen Bet v 1 or the corresponding protein from related tree species. Due to the low content of Ara h 8 in peanuts, sensitization to Ara h 8 is not always detectable using whole peanut extract [10]. Isolated Ara h 8 sensitization is almost always associated with peanut tolerance or mild symptoms upon peanut intake [11].

Patients with birch pollen-associated hazelnut allergy are sensitized to Cor a 1 and usually present with mild oral symptoms [12, 13]. IgE against the storage proteins Cor a 9 and Cor a 14, both major hazelnut allergens, can be used as markers for primary hazelnut sensitization and risk of more severe reactions [14, 15].

We have previously shown that sensitization to hazelnut and peanut is common among young asthmatics in Sweden [16]. Of the subjects included in this cohort, 54% were sensitized to hazelnut, 25% to peanut and 56% to birch pollen. Food allergen sensitization affects both local and systemic markers of inflammation in asthma [17, 18]. Patients with both asthma and food allergy have more severe asthma, with an increased risk of exacerbations, a higher rate of corticosteroid use and more frequent hospitalization, than asthmatics without food allergy [19–23]. Furthermore, asthma is a risk factor for fatal anaphylactic reactions to foods [24].

The aim of this study was to investigate molecular patterns of IgE antibody responses among young asthmatic individuals sensitized to peanut and hazelnut and to investigate correlations to levels of inflammation markers, exhaled NO, spirometry results, methacholine challenge and reported hypersensitivity reactions to these foods.

## Methods

### Study population

Within the framework of a Swedish academy-industry collaboration, the MIDAS project, a total of 408 children and young adults (10–≤ 18 respectively > 18–35 years of age) with physician-diagnosed asthma were recruited in Uppsala, Sweden [16, 17]. All subjects were on daily treatment with inhaled corticosteroids (ICS) and/or oral leukotriene receptor antagonists (LTRA) during at least 3 months of the year before study entry.

### IgE antibody measurements

All IgE analyses were performed by ImmunoCAP (Thermo Fisher Scientific, Uppsala, Sweden). IgE antibody concentrations ≥ 0.35 kU_A_/L were regarded as positive in this study. Peanut and hazelnut sensitization were defined as a positive IgE antibody test to any of whole peanut extract, Ara h 1, Ara h 2, Ara h 3 or Ara h 8 and to any of whole hazelnut extract, Cor a 1, Cor a 9 or Cor a 14 respectively.

Atopy was defined as a positive IgE test to a positive result from the multi-allergen food test fx(5) (egg, cow’s milk, cod fish, wheat, peanut, soy, hazelnut and shrimp) and/or a positive result from the multi-allergen test Phadiatop (grass, tree and weed pollen and animal, mite and mold). Fx(5) and Phadiatop were delivered by Thermo Fisher Scientific, Uppsala, Sweden,

### Measurement of airway and systemic inflammation markers and lung function

The fraction of exhaled nitric oxide (FeNO; airway inflammation marker) was measured with a chemiluminescence analyzer (NIOX Flex, Aerocrine AB, Solna, Sweden). Eosinophil blood count (B-Eos; systemic inflammation marker) was measured with a routine method (Cell-Dyn Sapphire, Abbott, Illinois, USA). Forced expiratory volume during one second (FEV1) was recorded with a MasterScope spirometer (Erich Jaeger, Wurzburg, Germany) and was used as a measure of pulmonary function. The methacholine provocative dose causing a fall of FEV1 by 20% (PD20) was determined with the Aerosol Provocation System (Viasys Healthcare GmbH, Germany). Measurements were done in accordance with standardized routines and guidelines [25–27] and have been described in detail elsewhere [16, 17, 28]. Blood samples used for ECP analysis (Eosinophil Cationic Protein, systemic inflammation marker) were collected in SST tubes and left for 60 min at room temperature. Thereafter, they were centrifuged at 3000 rpm for 15 min and stored in Sarstedt sample tubes at − 20 °C. The samples were analysed with the ImmunoCAP ECP assay. An S-ECP level ≥ 20 µg/L was defined as elevated.

### Asthma medication

The subjects’ use of ICS, combination ICS/long-acting beta-agonists (LABA) and/or LTRA during the past 3 months was recorded in the interview. The prescribed daily dose of ICS was collected from the subjects’ medical records [16].

### Perceived food hypersensitivity symptoms

Subjects were asked to report any history of hypersensitivity reactions upon intake of peanut and/or hazelnut during the last year. Symptoms were grouped according to the organ systems affected: the lower airways (asthma), the upper airways (rhinitis, conjunctivitis), the oral cavity (oral allergy syndrome), the skin (atopic dermatitis, urticarial, angioedema), the gastrointestinal tract (nausea, vomiting, stomach pain, diarrhea) and anaphylaxis (self-reported) [16]. Interviews were conducted by an allergy nurse, with the use of a structured questionnaire [16].

### Statistical analysis

Categorical variables were described using percentages and differences between groups were studied with the χ2 test. Normally distributed continuous variables were described using means and standard deviations and the *t* test was used to compare means. If continuous variables had a distribution skewed to the right (e.g. FeNO), a geometric mean with a 95% confidence interval was used for descriptive statistics and logarithm-transformation was performed before group comparisons. The PD20 was described using median and interquartile range and the Mann–Whitney test was used to compare groups [16]. Correlations between continuous numerical data were determined using Pearson’s correlation coefficient.

Data were analyzed using the statistics software package Stata (version 12; Stata Corporation, College Station, Texas, USA) and SAS (version 9.4, SAS, Cary, N.C., USA). p-values ≤ 0.05 were considered significant.

## Results

**Fig. 1:**
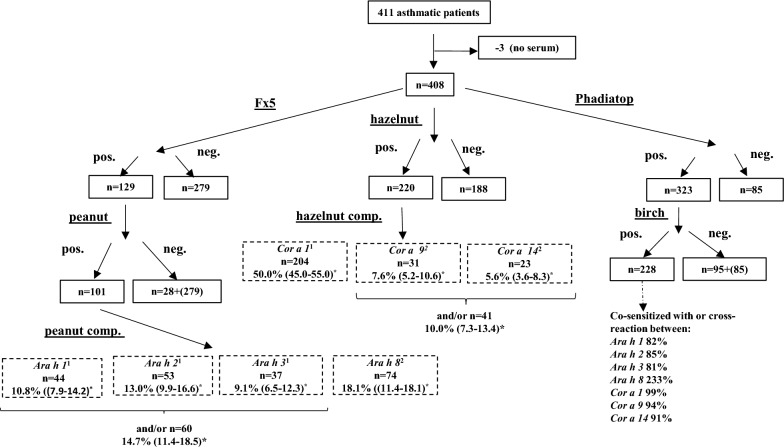
Specific IgE test chart for 408 asthmatic patients. Number of patients and *prevalence with 95% CI. ^1^A seed storage protein from peanut/hazelnut. ^2^A PR-10 protein hypothesized to be responsible for cross-reactivity between birch pollen and peanut/hazelnut

Of the 408 asthmatic patients included into the study, 200 were males and mean age was (± SEM) 20.4 ± 0.3 years. Sensitization to peanut and hazelnut was demonstrated in 101 (25%) and in 220 (54%) subjects, respectively, and sensitization to birch pollen was demonstrated in 228 (56%) subjects. Ninety-two (91%) peanut positive subjects were co-sensitized with hazelnut. In total 229 patients were additional tested for peanut and hazelnut related allergen components (Fig. 1, Table 1). Out of these, 215 were birch pollen positives.

**Table 1:** Sensitization rates for the studied population (n = 408)

	Number of positives (%)
Phadiatop (inhalant allergen mix)	323 (79)
fx5 (food allergen mix)	129 (32)
Hazelnut	220 (54)
Peanut	101 (25)
Birch	228 (56)

In addition to the 74 Ara h 8 positive patients among the 101 who were peanut positive, there were another 99 Ara h 8 sensitized patients without IgE antibodies to any of peanut storage proteins, suggesting the absence of primary peanut sensitization (99/215 = 46%). The situation for hazelnut sensitization was different. Nearly all hazelnut positives patients were also birch pollen positive. Amongst the birch pollen positive subjects, 183 (85%) were also positive to at least one of the peanut related allergen components and 206 (96%) to at least one of the hazelnut related allergen components.

### Airway and systemic inflammation markers in relation to allergen component sensitization

Subjects sensitized to any of Ara h 1, Ara h 2, Ara h 3, Cor a 9 or Cor a 14 were younger and had higher levels of FeNO, B-Eos, S-ECP and total IgE, than other subjects (Table 2, PH:3 vs PH:1). This group of patients also reported a higher frequency of eczema and they also had increased bronchial responsiveness (lower PD_20_) than patients not sensitized to these seed storage proteins. The findings regarding the levels of FeNO, B-Eos, S-ECP and PD_20_ persisted after adjustments for age, sex, weight, height, FEV_1_, current dose of ICS and current smoking (data not shown). However, after further adjustments for presence of aeroallergen sensitization, subjects sensitized to peanut and/or hazelnut storage proteins still had higher levels of FeNO, B-Eos and S-ECP whilst the association to PD20 became statistically non-significant (data not shown).

**Table 2:** Patient characteristics and inflammation markers in relation to allergen component sensitization (n = 408)

	Peanut and Hazelnut in any combination PH:1 PH:2 PH:3			Peanut P:1 P:2 P:3			Hazelnut H:1 H:2 H:3		
Number (%)	179 (44)	156 (38)	73 (18)	207 (51)	141 (34)	60 (15)	188 (46)	179 (44)	41 (10)
Age (years)^a^	20.9 ± 0.5	21.2 ± 0.6	17.6 ± 0.7*^,#^	21 ± 0.5	21 ± 0.6	17.2 ± 0.7^¤,#^	20.8 ± 0.5	20.5 ± 0.5	17.9 ± 0.9
Female^b^	95 (53)	83 (53)	30 (41)	107 (52)	77 (55)	24 (40)	101 (54)	90 (50)	17 (41)
FeNO (ppb)3	12.7 (11.3–14.2)	16.7 (14.8–18.7)*	23.2 (19.6–27.5)*^,#^	13.1 (11.7–14.5)	17.3 (15.4–19.5)^¤^	23.6 (19.5–28.5)^¤,#^	12.8 (11.5–14.3)	17.7 (15.8–19.9)^∆^	23.5 (19.1–29)^∆^
FEV1 (%)1	92.9 ± 1.1	92.2 ± 1.2	89.6 ± 1.6	93 ± 1	91.8 ± 1.2	89.4 ± 1.8	92.9 ± 1	91.9 ± 1	89 ± 2.4
Methacholine ch.test (PD20)^d^	0.4 (0.1–3)	0.3 (0.1–1.8)	0.1 (0.1–0.4)*	0.4 (0.1–2.9)	0.3 (0.1–1.2)	0.1 (0–0.1)	0.4 (0.1–7.2)	0.3 (0.1–1.2)	0.1 (0–0.6)
Eosinophils (10^9^/L)^c^	0.15 (0.13–0.16)	0.17 (0.15–0.2)	0.34 (0.29–0.41)*^,#^	0.14 (0.13–0.16)	0.19 (0.17–0.22)^¤^	0.34 (0.28–0.41)^¤,#^	0.15 (0.13–0.17)	0.19 (0.17–0.22)^∆^	0.37 (0.29–0.46)^∆,☼^
ECP (µg/L)^c^	11.6 (10.5–12.7)	12.4 (11.1–13.8)	20 (17–23.5)*^,#^	11.6 (10.6–12.7)	13.2 (11.7 –14.9)	19.7 (16.5–23.5)^¤,#^	11.7 (10.6–12.8)	13.1 (11.8–14.5)	22.1 (17.8–27.4)^∆,☼^
Total IgE (kU/L)^c^	58 (46–73)	213 (176–258)*	523 (396–689)*^,#^	63 (51–78)	265 (216–325)^¤^	515 (378–701)^¤,#^	62 (49–77)	226 (190–270)^∆^	803 (557–1158)^∆,☼^
Eczema (ever)^b^	67 (37)	100 (64)*	59 (81)*^,#^	77 (37)	101 (72)^¤^	48 (80)^¤^	73 (39)	118 (66)^∆^	35 (85)^∆,☼^
Inh.corticosteroids (µg/day)^c^	404 (369–443)	416 (375–461)	358 (315–407)	400 (369–434)	425 (380–475)	345 (299–399)	404 (370–442)	404 (367–444)	368 (307–442)
Birch pollen sensitization^b^	13 (7)	151 (97) *	64 (88)*^,#^	39 (19)	137 (97) ^¤^	52 (87)^¤,#^	14 (7)	176 (98)^∆^	38 (93)^∆^
Ara h 8 sensitization^b^				–	132 (94)	42 (70)^#^			
Cor a 1 sensitization^b^							–	169 (94)	35 (85)

*PH:1* not sensitized to peanut or hazelnut; *PH::2* sensitized to peanut and/or Ara h8 and/or hazelnut and/or Cor a 1 but not to any of Ara h 1, 2, 3, Cor a 9, 14; *PH::3* sensitized to peanut and/or Ara h8 and/or hazelnut and/or Cor a and to any of Ara h 1, 2, 3, Cor a 9, 14

*P:1* not sensitized to peanut; *P:2* sensitized to peanut but not to any of Ara h 1, 2, 3; *P:3* sensitized to peanut and to any of Ara h 1, 2, 3

*H:1* not sensitized to hazelnut; *H:2* sensitized to hazelnut but not to any of Cor a 9, 14; *H:3* sensitized to hazelnut and to any of Cor a 9, 14

^a^Mean ± SD, ^b^Number (percentage), ^c^Geometric Mean (95% CI), ^d^median (IQR)

* p < 0.05 compared to group A1

^#^p < 0.05 compared to group A2

^¤^p < 0.05 compared to group B1

^##^p < 0.05 compared to group B2

^∆^p < 0.05 compared to group C1

^☼^p < 0.05 compared to group C2

Higher levels of FeNO, ECP and B-Eos were found in subjects sensitized to peanut and any of Ara h 1, 2 and 3 as compared to peanut non-sensitized subjects (Table 2, P:3 vs P:1; Fig. 2). Furthermore, subjects sensitized to peanut, but not to any of Ara h 1, 2 and 3 (Table 2, P:2 vs P:1; Fig. 2) had higher levels of FeNO and B-Eos compared with subjects not sensitized to peanut. Peanut-sensitized subjects without sensitization to Ara h 1, Ara h 2 or Ara h 3 were more frequently sensitized to Ara h 8 and birch pollen than those with sensitization to peanut storage proteins (Fig. 1).

**Fig. 2:**
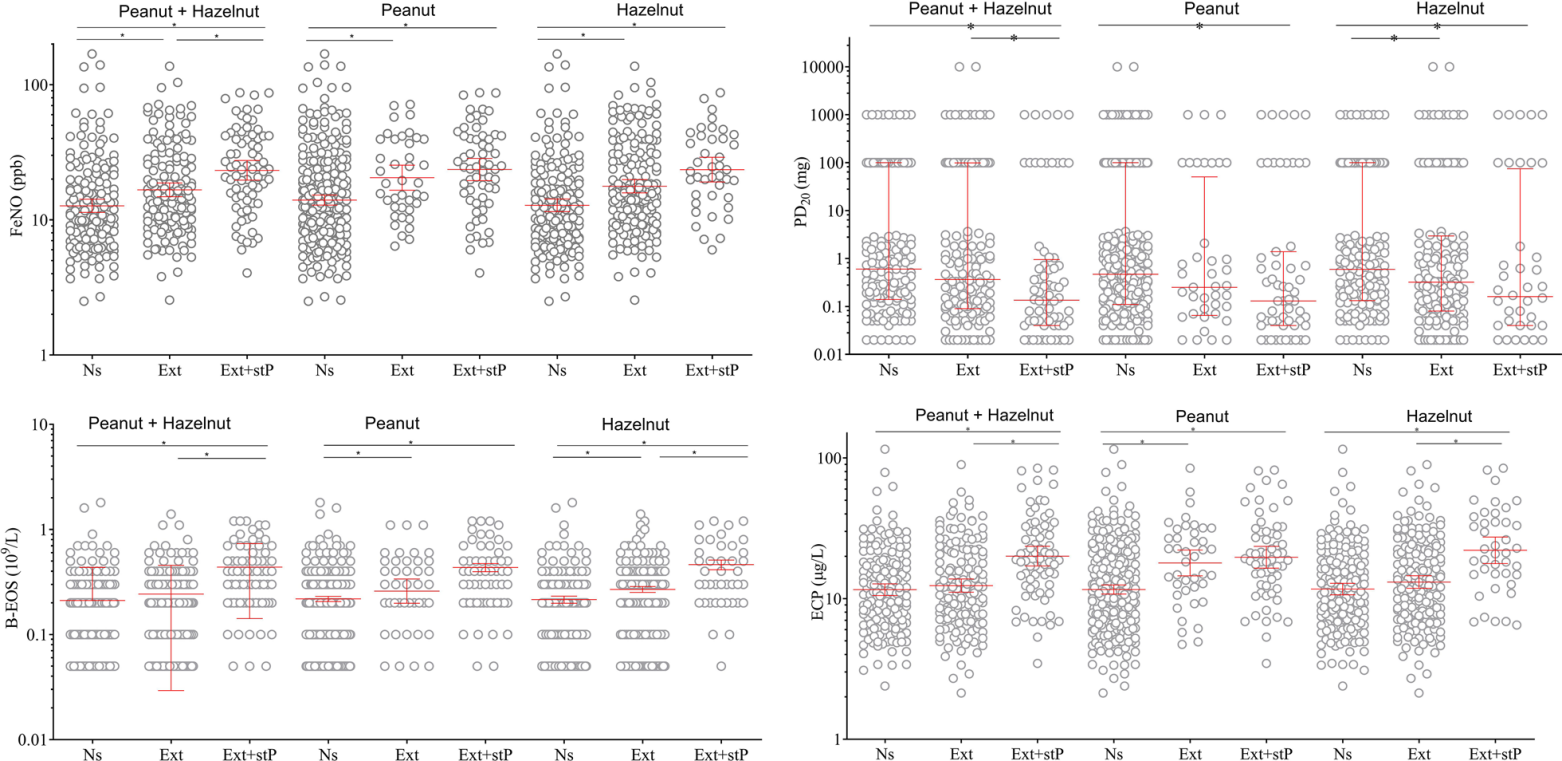
FeNO (upper left panel), PD20 (upper right panel), B-Eos and ECP in different groups of asthmatic subjects, stratified with respect to sensitization to peanut and/or hazelnut extract, and corresponding storage proteins, respectively. Figures depict GM (95% CI) for all variables except PD20, depicted as median (IQR). *p-value < 0.05. *Ns* not sensitized to extract, *Ext* sensitized to extract only, *Ext* + *stP* sensitized to extract and storage proteins

Higher levels of ECP and B-Eos were found in subjects sensitized to hazelnut and any of Cor a 9 and Cor a 14 as compared to hazelnut non-sensitized subjects (Table 2, H:3 vs H:1; Fig. 2). Furthermore, subjects sensitized to hazelnut, but not to Cor a 9 or Cor a 14, had higher levels of FeNO and B-Eos compared with subjects not sensitized to hazelnut (Table 2, H:3 vs H:2; Fig. 2). Hazelnut-sensitized subjects without sensitization to Cor a 9 or Cor a 14 did not differ in frequency of sensitization to Cor a 1 and birch pollen, when compared to those with sensitization to hazelnut storage proteins (Fig. 1).

### IgE antibody levels in relation to airway and systemic inflammation markers in children and adults

Among peanut sensitized subjects, sensitization to any of Ara h 1, Ara h 2 or Ara h 3 was more common in those below 18 years of age (20% vs. 10% in adult subjects, p < 0.001). Such a relationship with age was also true for hazelnut-sensitized subjects, where sensitization to any of Cor a 9 or Cor a 14 was more common in subjects younger than 18 years (14% vs. 7% in adult subjects, p < 0.05). The frequency of atopy did not differ between children and adults (80% vs. 81%).

Levels of IgE antibodies to all allergen components except for Ara h 2 and Cor a 1 were higher in children than in adults (Table 3).

**Table 3:** Comparison of IgE antibody levels against peanut and hazelnut allergen components between children and adults

	Ara h 1	Ara h 2	Ara h 3	Ara h 8	Cor 1	Cor a 9	Cor a 14
All	0.13 (< 0.1–0.18)	0.21 (0.12–0.37)	< 0.1(< 0.1– < 0.1)	1.2 (0.94–1.6)	4.4 (3.4–5.7)	< 0.1(< 0.1–< 0.1)	< 0.1(< 0.1–< 0.1)
Children	0.20 (0.13–0.33)	0.27 (0.12–0.58)	< 0.1 (< 0.1–0.17)	1.9 (1.3–2.8)	5.7 (3.9–8.3)	0.11 (< 0.1–0.20)	< 0.1(< 0.1–0.15)
Adults	< 0.1 (< 0.1–0.13)**	0.15 (< 0.1–0.35)	< 0.1(< 0.1 – < 0.1)*	0.85 (0.58–1.3)**	3.4 (2.4–4.9)	< 0.1(< 0.1– < 0.1)**	< 0.1(< 0.1– < 0.1)***

* p < 0.05, ** p < 0.01, *** p < 0.001

Among the children, statistically significant correlations were found between levels of FeNO and levels of IgE antibodies against Ara h 1, Ara h 2, Ara h 3, Cor a 9 and Cor a 14, but not against Ara h 8 or Cor a 1 (Table 4). No such correlation between FeNO and IgE antibody levels was found among the adult subjects. Levels of B-Eos correlated with levels of IgE antibodies against all allergen components investigated among the children and with IgE antibodies against the peanut and hazelnut storage proteins among the adults.

**Table 4:** Correlation between IgE antibody levels and FeNO (A) and B-Eos (B), for all subjects, children and adults

	Ara h 1	Ara h 2	Ara h 3	Ara h 8	Cor 1	Cor a 9	Cor a 14
(A) IgE level and FeNO							
All subjects	0.24***	0.25***	0.27***	0.05	0.07	0.25**	0.29***
Children	0.29**	0.31**	0.32***	0.03	0.05	0.27**	0.36***
Adults	0.14	0.14	0.16	0.02	0.04	0.17	0.14
(B) IgE level and B-EOS							
All subjects	0.35***	0.38***	0.40***	0.23***	0.19**	0.34***	0.36***
Children	0.38***	0.46***	0.50***	0.21**	0.12	0.36***	0.38***
Adults	0.25***	0.22**	0.21*	0.17	0.17	0.22*	0.22*

* p < 0.05, ** p < 0.01, ***p < 0.001

### Reported food hypersensitivity in relation to allergen component sensitization

Hypersensitivity symptoms upon peanut intake were more frequently reported among subjects sensitized to any of Ara h 1, Ara h 2 or Ara h 3 than among all other subjects (Table 5, P:3). Hypersensitivity symptoms upon hazelnut intake were more frequently reported among subjects sensitized to hazelnuts, regardless of sensitization to Cor a 9 or Cor a 14, than among those not sensitized to hazelnut (Table 5, H:2, H:3 vs H:1). Those sensitized to Cor a 9 or Cor a 14 were, however, less likely to report absence of symptoms upon hazelnut intake.

**Table 5:** Reported hypersensitivity symptoms and type of symptoms in relation to peanut and hazelnut sensitization profiles

	Peanut^a^ P:1 P:2 P:3			Hazelnut^b^ H:1 H:2 H:3		
	n = 207	n = 141	n = 60	n = 188	n = 179	n = 41
No symptoms	186 (90)	87 (62)*	2 (4)*^,#^	160 (85)	74 (41)*	4 (9)*^,#^
Uncertain/never tried peanuts	4 (2)	26 (18)*	14 (23)*	6 (3)	32 (18)*	15 (37)*^,#^
Yes	17 (8)	28 (20)*	44 (73)*^, #^	22 (12)	73 (41)*	22 (54)*
Lower airway symptoms	9 (53)	13 (46)	31 (70)	11 (50)	25 (34)	11 (50)
Upper airway symptoms	1 (6)	4 (14)	5 (11)	3 (14)	6 (8)	2 (9)
Oral cavity symptoms	11 (65)	24 (86)	26 (59)^#^	16 (73)	63 (86)	19 (86)
Skin symptoms	2 (12)	5 (18)	22 (50^)^ *^,#^	3 (14)	17 (23)	9 (41)*
Gastrointestinal symptoms	1 (6)	4 (14)	12 (27)	1 (5)	5 (7)	10 (45)*^,#^
Anaphylaxis	2 (12)	1 (4)	10 (23) ^#^	2 (9)	2 (3)	3 (14)^#^

^a^*P:1* not sensitized to peanut; *P:2* sensitized to peanut/Ara h 8 but not to any of Ara h 1, 2, 3; *P:3* sensitized to peanut and to any of Ara h 1, 2, 3

^b^*H:1* not sensitized to hazelnut; *H:2* sensitized to hazelnut/Cor a 1 but not to any of Cor a 9, 14; *H:3* sensitized to hazelnut and to any of Cor a 9, 14

* p < 0.05 compared to group P:1 respectively H:1

^#^p < 0.05 compared to group P:2 respectively H:2

Subjects sensitized to any of Ara h 1, Ara h 2 or Ara h 3 more frequently reported skin symptoms upon peanut intake than all other subjects. (Table 5, P:3). The same group also reported a higher frequency of anaphylaxis and a lower frequency of symptoms from the oral cavity than other peanut sensitized subjects. Subjects sensitized to Cor a 9 or Cor a 14 more frequently reported gastrointestinal symptoms upon hazelnut intake than subjects who were sensitized to hazelnut and/or Cor a 1 but not to Cor a 9 or Cor a 14 (Table 5, H:3 vs H:2).

## Discussion

In this population of young asthmatics, sensitization to peanut and hazelnut storage proteins was found to be more common in children than in adults and was related both to local and systemic inflammation. Further, a correlation between levels of FeNO and IgE antibodies against peanut and hazelnut storage proteins was observed in children but not in adults.

To our knowledge, this is the first time an asthma cohort consisting of young individuals has been examined regarding patterns of food allergen component sensitization and inflammation markers. We believe that our findings may have clinical implications. In patients sensitized to peanut and hazelnut storage proteins, avoidance of these foods is of importance not only because of the risk of food allergic reactions but also because of an increased risk of respiratory symptoms. A thorough investigation of these asthmatic individuals should be performed, including food challenge if needed. If the peanut sensitization is due to pollen cross-reaction, strict avoidance is not necessary. Thus, one conclusion that can be drawn is that asthmatic patients avoiding nuts due to reactions upon intake should be investigated for sensitization to relevant nut allergen components.

Nut storage proteins, especially the 2S albumins, are becoming of increasing interest in nutritional and clinical studies, as they have been reported as major food allergens [29] Although the basis of their allergenic potency is not fully understood, stability to food processing and high resistance against pepsin, trypsin and chymotrypsin digestion are considered to be important factor, in addition to their abundance. Our findings show a correlation between sensitization against storage proteins and inflammatory markers, which further supports the need to identify sensitization to these proteins as indicators of disease processes and an elevated risk of severe allergic reactions. The need of good asthma control in patients with sensitization to these proteins is also emphasized, in particular with respect to the proper use of corticosteroids and other anti-inflammatory treatments.

The reason for stronger signs of inflammation in asthmatic patients with than without sensitization to nut storage proteins is presently unclear. Given that most patients sensitized to the storage proteins are likely to avoid peanuts and tree nuts, it appears unlikely that inflammation is maintained by ongoing subclinical IgE-mediated allergic reactions. We propose that this is more likely a bone marrow derived primary cytokine driven phenomenon that in parallel causes both sensitization to strong allergens and signs of Th2-related inflammation [30–32].

The difference between children and young adults regarding the correlation between sensitization and levels of FeNO is an interesting observation. This finding was unexpected and has to our knowledge not previously been described. One possible interpretation is that allergic children in general are both more immunologically active and reactive than allergic adults. Another explanation could be that this is a cohort effect and that the children born in the twenty-first century have more nut allergies compared to an older generation. A third possibility could be tolerance development in the young adults compared to children. Our findings need to be independently corroborated by studies in other patient populations.

Food allergy is related to more severe asthma disease, with an increased risk of exacerbations, a higher rate of corticosteroid use, and more frequent hospitalizations [21, 33]. Conversely, the prevalence of food allergy appears to be higher among asthmatic subjects than in the general population. It is difficult for the practicing physician to make a risk assessment of an asthmatic patient with suspected peanut or tree nut allergy. The assessment is usually based on symptoms, eliciting dose and a sensitization test based on whole food extract. Dua et al. have shown that exercise and sleep deprivation each significantly reduce the threshold of reactivity in patients with peanut allergy [34]. It seems that estimating the risk of future peanut reaction based on eliciting dose of ingested peanut is difficult. Further, IgE levels of whole allergen such as peanut and hazelnut is difficult to interpret, especially in the presence of birch pollen sensitization due to cross-reactivity between Bet v 1 and homologous food allergens. We are able to show in this study that being sensitized to nut storage proteins is associated with a higher risk of systemic and local inflammation as compared to being sensitized to a Bet v 1 homologue, such as Ara h 8 or Cor a 1. In order to make an adequate risk assessment, asthma patients experiencing symptoms upon nut intake should therefore be tested for sensitization to relevant nut storage proteins.

There was no association between storage protein sensitization and lower airway symptoms upon peanut or hazelnut intake in our study. This is in contrast to earlier studies, where children sensitized to peanut storage proteins more frequently reported symptoms from the lower airways upon peanut intake than children sensitized only to Ara h 8 [10]. One possible explanation for this is that our study population only included subjects with asthma and that these subjects in general are more likely to report respiratory symptoms from the lower airways. Another explanation could be that these patients are careful regarding the intake of allergenic food such as peanuts and nuts and therefore seldom experience food-induced respiratory symptoms.

A strength of our study is the detailed information regarding both asthma morbidity and sensitization patterns in a population known to be at high risk of severe allergic reactions [24]. A limitation of the study is that we lack food challenge data in order to objectively confirming food hypersensitivity. However other studies, such as Andorf et al. have recently shown the associations between Cor a 9 and Cor a 14 sensitization and DBPCFC outcomes [35]. This limitation that we did not perform food challenge in this cohort of asthmatics has been discussed in detail elsewhere [16].

## Conclusions

We conclude that a subgroup of young asthmatic individuals displays sensitization to peanut and hazelnut storage proteins. Storage protein sensitization appears to be associated with asthma morbidity, as this group has higher levels of both local and systemic inflammation markers.

## Data Availability

The authors confirm that all data underlying the findings are fully available without restriction. All relevant data are within the paper.

## Abbreviations

B-Eos: Blood eosinophil counts
ECP: Eosinophil cationic protein
FeNO: Fraction of exhaled nitric oxide
FEV_1_: Forced expiratory volume in one second
ICS: Inhaled corticosteroids
LTRA: Leukotriene receptor antagonists
MIDAS: Minimally-Invasive Diagnostic procedures in allergy, ASthma, or food hypersensitivity
PD_20_: The methacholine provocative dose causing a fall of FEV_1_ by 20%
